# Are There Rab GTPases in Archaea?

**DOI:** 10.1093/molbev/msw061

**Published:** 2016-03-31

**Authors:** Jaroslaw Surkont, Jose B. Pereira-Leal

**Affiliations:** ^1^Instituto Gulbenkian de Ciencia, Oeiras, Portugal

**Keywords:** eukaryogenesis, Lokiarchaeum, endomembrane trafficking system, Ras, LECA, protein prenylation.

## Abstract

A complex endomembrane system is one of the hallmarks of Eukaryotes. Vesicle trafficking between compartments is controlled by a diverse protein repertoire, including Rab GTPases. These small GTP-binding proteins contribute identity and specificity to the system, and by working as molecular switches, trigger multiple events in vesicle budding, transport, and fusion. A diverse collection of Rab GTPases already existed in the ancestral Eukaryote, yet, it is unclear how such elaborate repertoire emerged. A novel archaeal phylum, the Lokiarchaeota, revealed that several eukaryotic-like protein systems, including small GTPases, are present in Archaea. Here, we test the hypothesis that the Rab family of small GTPases predates the origin of Eukaryotes. Our bioinformatic pipeline detected multiple putative Rab-like proteins in several archaeal species. Our analyses revealed the presence and strict conservation of sequence features that distinguish eukaryotic Rabs from other small GTPases (Rab family motifs), mapping to the same regions in the structure as in eukaryotic Rabs. These mediate Rab-specific interactions with regulators of the REP/GDI (Rab Escort Protein/GDP dissociation Inhibitor) family. Sensitive structure-based methods further revealed the existence of REP/GDI-like genes in Archaea, involved in isoprenyl metabolism. Our analysis supports a scenario where Rabs differentiated into an independent family in Archaea, interacting with proteins involved in membrane biogenesis. These results further support the archaeal nature of the eukaryotic ancestor and provide a new insight into the intermediate stages and the evolutionary path toward the complex membrane-associated signaling circuits that characterize the Ras superfamily of small GTPases, and specifically Rab proteins.

## Introduction

A major question in evolutionary biology is the origin of the Eukaryotic cell plan, which is characterized by a multitude of intracellular organelles, including the energy producing endosymbiotic organelles, complex endomembrane trafficking system, and a nucleus containing a large genome that encodes thousands of genes. The protein repertoires associated with these organelles have been found in most Eukaryotes, suggesting that they were already present in the Last Eukaryotic Common Ancestor (LECA) (e.g., Field and Dacks 2009; Schlacht et al. 2014). Like in other areas of the evolutionary biology, the search for intermediate, transitional forms has attracted the attention of many, and eukaryotic-like cellular features or gene repertoires have been identified in different prokaryotes, for example, having been termed as the “dispersed eukaryome” in Archaea (Koonin and Yutin 2014).

Inferring ancient events such as the origin of Eukaryotes or the origin of their specific molecular traits is a very challenging task given the timescale, data scarcity, and insufficient methods. Despite this, mounting evidence suggests that the ancestral host cell that accommodated the endosymbiotic bacteria, which gave rise to mitochondria, was from the archaeal lineage (Lake et al. 1984; Cox et al. 2008, reviewed in López-García and Moreira 2015). This host cell may have in fact evolved from within Archaea (the TACK superphylum), rather than result from a much earlier branching as a sister group to all Archaea (Guy and Ettema 2011; Kelly et al. 2011; Williams et al. 2012, 2013; Williams and Embley 2014; Raymann et al. 2015). This scenario suggests that the search for transitional states should be carried out within the archaeal domain, and specifically the TACK superphylum.

A recent metagenomic survey of a deep ocean sediment sample from the Arctic Mid-Ocean Ridge revealed the existence of a new archaeal phylum within the TACK superphylum, the Lokiarchaeota (Spang et al. 2015). The authors reported that several building blocks characteristic of Eukaryotes are present in this taxon, suggesting that Lokiarchaeota and Eukaryotes share a common ancestor and that Lokiarchaeota is a modern descendant of that ancestor. Small GTPase gene families are highly expanded in Lokiarchaeota compared with other Archaea, including many small GTPases from the RAS superfamily; they form several distinct clusters, yet their relationship to the eukaryotic GTPases remains unclear.

The eukaryotic RAS superfamily contains five major families Arf, Ras, Rho, Ran, and Rab that are involved in the intracellular signaling and share the common G domain core (GTPase activity), responsible for the switching mechanism between the GTP-bound active and GDP-bound inactive state. The Arf family is involved in regulation of vesicular transport, Ras in response to diverse extracellular stimuli, Rho in actin dynamics, and Ran in nucleocytoplasmic transport (reviewed in Wennerberg et al. 2005). Here, we focused on Rab GTPases, critical regulators of vesicular trafficking systems (Fukuda 2008; Stenmark 2009; Kelly et al. 2012; Pfeffer 2013), included in the list of eukaryotic signature proteins, that is, “proteins that are found in eukaryotic cells but have no significant homology to proteins in Archaea and Bacteria” (Hartman and Fedorov 2002). This family has experienced extensive universal and taxon-specific duplications associated with the emergence of major organelles and organelle specializations of the endomembrane system; each Rab subfamily provides specificity to a particular component of the trafficking system and this function is generally conserved throughout evolution (Dacks and Field 2007; Dacks et al. 2009; Brighouse et al. 2010; Diekmann et al. 2011). They form the largest RAS family, with more than 60 Rab homologues in human (Pereira-Leal and Seabra 2001), and several studies point to the existence of a rich Rab repertoire at the LECA (Diekmann et al. 2011; Elias et al. 2012; Klöpper et al. 2012); however, they have been so far restricted to the eukaryotic domain. Here, we test the hypothesis that Rab GTPases predate Eukaryogenesis, by investigating the small GTPase repertoire in Archaea, and in particular the expanded small GTPase family in the recently described Lokiarchaea.

## Results

### Multiple Rab-like Sequences in Archaea

**Fig. 1:**
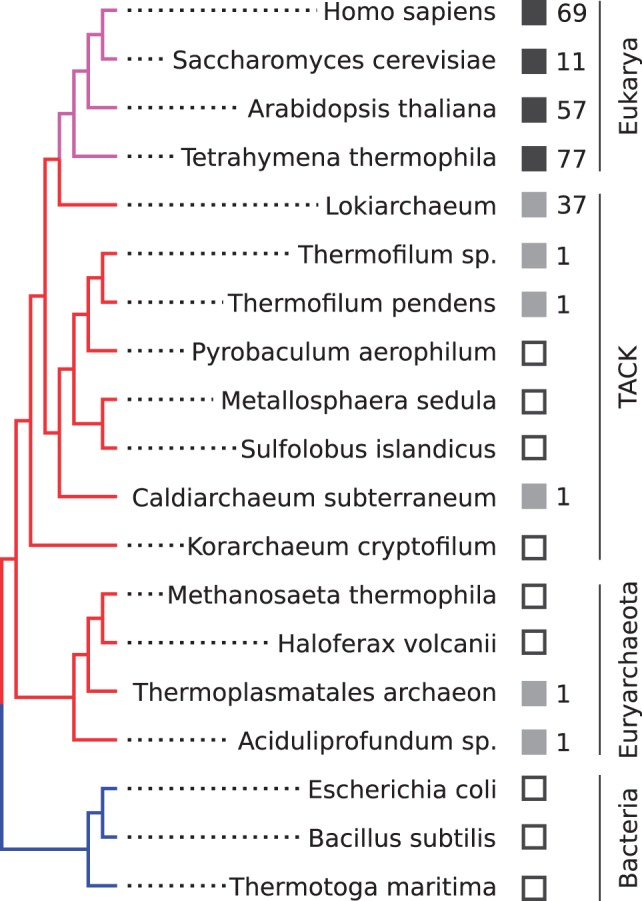
Phylogenetic profile of the Rab family in representative species of eukaryotes (magenta), Archaea (red), and bacteria (blue). The remaining archaeal species that were used in the analysis, without Rab-like protein predictions, are not shown in the figure. A full (hollow) square indicates the presence (absence) of at least one predicted eukaryotic Rab protein (black) or archaeal Rab-like protein (gray). The total number of Rab homologues is shown next to the square. TACK refers to the superphylum that comprises the Thaumarchaeota, Aigarchaeota, Crenarchaeota, and Korarchaeota phyla. Tree topology is consistent with Spang et al. (2015).

In the original metagenomic study by Spang et al. (2015) the assembly of a complete archaeal genome defined a novel archaeal phylum, the Lokiarchaeota. In this Lokiarchaeum genome, more than 90 members of the RAS superfamily were predicted, yet it is unclear whether these proteins belong to any specific, previously described RAS family or constitute a novel group. Here, we systematically searched all complete archaeal genomes, including the Lokiarchaeum, for members of the RAS superfamily of small GTPases and specifically annotated Rab-like proteins. We used the Rabifier (Diekmann et al. 2011), a bioinformatic pipeline that runs a series of consecutive classification steps as follows: 1) determining if a protein contains the small GTPase domain, 2) whether it belongs to the Rab family or another member of the RAS superfamily, and 3) what is the most likely Rab subfamily assignment of the protein. We detected a total of 3,152 proteins containing the small GTPase domain, of which 133 within the Lokiarachaeum genome (the remaining an average of 13.6 ± 3.4 proteins per genome). Of this total, 42 were predicted as Rab-like GTPases without any specific subfamily annotation, that is, none of the Rab-like proteins is sufficiently similar to any of the established eukaryotic subfamilies. Among the 42 Rab-like proteins 37 belong to Lokiarchaeum, the remaining five (one copy per species) were identified in *Thermofilum pendens*, *Thermofilum* sp., *Caldiarchaeum subterraneum*, *Thermoplasmatales archaeon*, and *Aciduliprofundum* sp. These species are distributed across Archaea, they belong to one of two major superphyla, Euryarchaeota and TACK. This raises a question about the origin of these Rab-like proteins, as their phylogenetic profile (fig. 1) does not reveal any obvious pattern of vertical inheritance.

### Inconclusive Phylogenetic Positioning of Archaeal Rab-like Sequences

Our bioinformatic analysis confirms the presence of many small GTPases in Archaea and identifies multiple Rab-like GTPases in diverse archaeal species, yet without any subfamily assignment. To determine the position of archaeal Rab-like proteins within the superfamily of small GTPases and their relationship to eukaryotic Rabs, we conducted a phylogenetic analysis of archaeal Rab-like proteins together with the eukaryotic Rabs which are likely present in the LECA (Diekmann et al. 2011; Elias et al. 2012), also including representative sequences of other RAS families. We used both Bayesian and Maximum Likelihood approaches for the phylogenetic inference (see Materials and Methods for details).

**Fig. 2:**
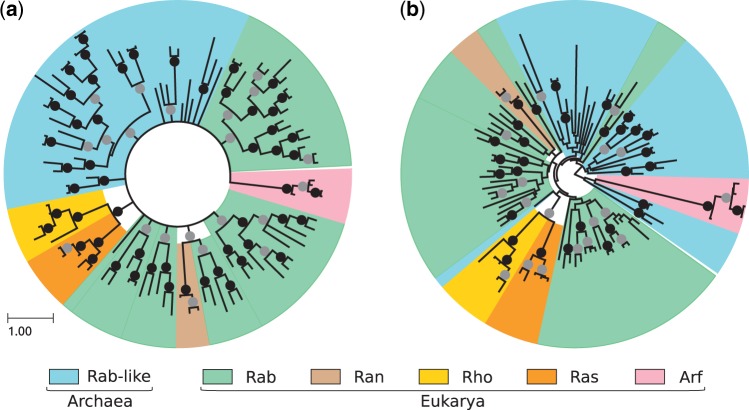
Phylogeny of small GTPases from Eukarya and Archaea using (*a*) Bayesian and (*b*) maximum-likelihood inference. Representative eukaryotic members of all RAS families (Rab, Ran, Rho, Ras, and Arf) and putative archaeal Rab-like are included. Black (gray) circle indicates a Bayesian posterior probability value above 0.9 (0.6) and a bootstrap support value above 90 (60) for a branch split. Branch lengths are proportional to the expected number of substitutions per site, as indicated by the scale bar.

As previously observed (Dong et al. 2007; Rojas et al. 2012), trees of small GTPases have very weak statistical support for basal branches (Rho vs. Rab vs. Ras, etc.), and Rabs may appear in multiple independent basal branches (fig. 2, supplementary fig. S1, Supplementary Material online). Archaeal Rab-like sequences are monophyletic with the eukaryotic proteins, indicating that they are more similar to sequences from Eukaryotes than to other small GTPases from Archaea (supplementary fig. S2, Supplementary Material online). They are however not monophyletic with any one specific small GTPase family, being part of a basal polytomy (fig. 2).

To gain a more detailed view on the Rab-like family structure, we constructed a phylogenetic tree using only archaeal Rab-like sequences (supplementary fig. S3, Supplementary Material online). Although the deep branching pattern could not be reliably resolved, we observed that most of the sequences cluster within several highly supported groups. Short terminal branches suggest recent duplication of several Lokiarchaean proteins. Proteins from *Thermoplasmatales*, *Aciduliprofundum*, and *Caldiarchaeum* form long branches indicating very divergent sequences, which do not cluster together with Lokiarchaeum. In contrast, proteins from both *Thermofilum* species form a distinct cluster with two other Lokiarchaean sequences.

Overall, this analysis suggests that phylogenetic methods alone are insufficient to determine the relationship between archaeal Rab-like GTPases and the eukaryotic members of the RAS superfamily. This, however, raises the question of why these sequences were classified as Rab-like.

### Rab-like Proteins Contain Typical Eukaryotic Rab Motifs

We next analyzed sequence properties of archaeal Rab-like GTPases at the family level to further assess their similarity to other members of the RAS superfamily. We constructed a sequence model for each family (Rab, archaeal Rab-like, Ran, Rho, Ras, Arf). We first built multiple sequence alignments using representative sequences for each family and a seed alignment of the small GTPase domain (Pfam:PF00071) to guide the alignment process and improve an overall quality of the alignment, the seed sequences were then removed from the final alignment. The alignments were subsequently used to construct profile hidden Markov models (pHMMs) and generate plurality-rule consensus sequences that describe each family.

We first calculated the overall, pairwise similarity between the families (supplementary table S2, Supplementary Material online) and observed a remarkable similarity of 78% (60% identity, local alignment) between eukaryotic Rab and archaeal Rab-like GTPases (71% and 55%, respectively, for global alignment, supplementary table S3, Supplementary Material online), much higher than between the archaeal Rab-like family and any other member of the RAS superfamily. We subsequently focused on a more specific comparison between Rab-like and Rab proteins; we compared amino acid variation along the sequence across Rab paralogues in *Lokiarchaeum* and representative species from different major eukaryotic groups (*Homo* *sapiens*, *Trypanosoma* *brucei*, and *Guillardia* *theta*). We observed similar patterns of variation for all analyzed species (supplementary fig. S4, Supplementary Material online): regions of both low and high sequence conservation belong to the corresponding positions in the Rab sequences from different species, suggesting that archaeal Rab-like sequences are evolutionarily constrained in the same regions as the eukaryotic Rabs.

**Fig. 3:**
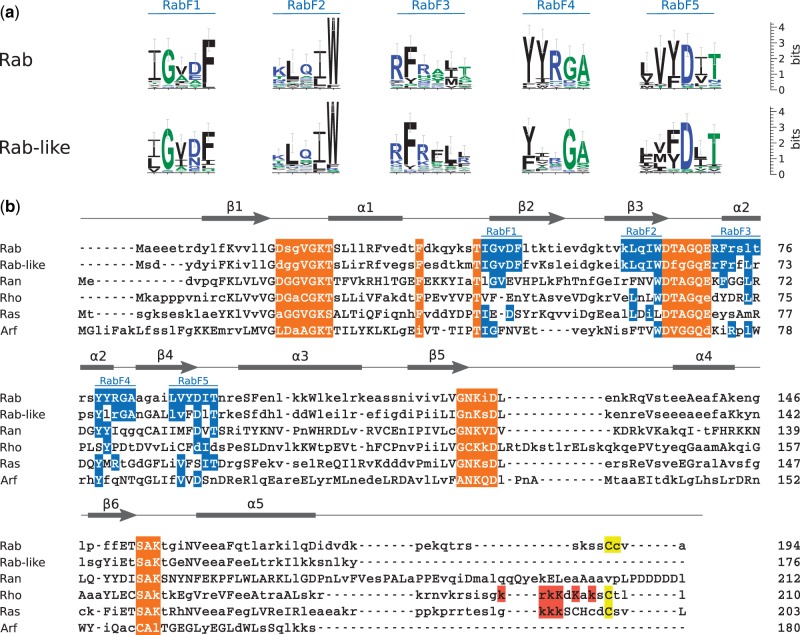
Sequence comparison of small GTPase families. (*a*) Sequence logo comparison of RabF motifs between Rab and Rab-like families. (*b*) Alignment of the consensus sequences generated with pHMMs of the eukaryotic RAS families and the archaeal Rab-like family. RabF motifs in the Rab family and identical residues at the corresponding positions in other families are highlighted in blue. Orange highlight denotes the guanine nucleotide-binding positions. Red indicates positively charged C-terminal amino acids. Yellow indicates the C-terminal cysteines, which are often posttranslationally modified. Upper case indicates residues with probability greater than 0.5 in the HMM profile. Secondary structure elements are denoted by bars (*α*-helices) and arrows (*β*-sheets).

We next tested the hypothesis that sequence conservation between archaeal and eukaryotic sequences is associated with the RabF motifs—sequence motifs unique to the Rab family that are important for the interaction with Rab effectors (Pereira-Leal and Seabra 2000). The results of this analysis are summarized in figure 3. All positions that correspond to the RabF1 and RabF2 motifs in eukaryotic Rabs are conserved in the archaeal Rab-like sequence. For comparison, in other families at most two amino acids are conserved at the corresponding positions. In the remaining three motifs most of the residues are identically conserved between Rab and Rab-like sequences, some are similar, for example, positively charged arginine and lysine in RabF4, aliphatic isoleucine and leucine in RabF5, and aromatic tyrosine and phenylalanine in RabF5 (tyrosine is also the second most common amino acid at this position in the archaeal sequences). From the sequence perspective, archaeal Rab-like proteins have all the hallmarks of Rabs, including the motifs involved in binding Rab regulators and effectors.

The major difference between eukaryotic Rab and archaeal Rab-like sequences is the absence of C-terminal cysteine residues, the prenylation sites of the eukaryotic Rabs, in all of the analyzed archaeal sequences. Rab-like sequences tend to have a shorter C-terminal sequence, missing most of what is termed the (flexible) hypervariable region in eukaryotic Rabs, known to be involved in associations with the membrane.

### Rab-like Proteins Are Structurally Similar to Eukaryotic Rabs

**Fig. 4:**
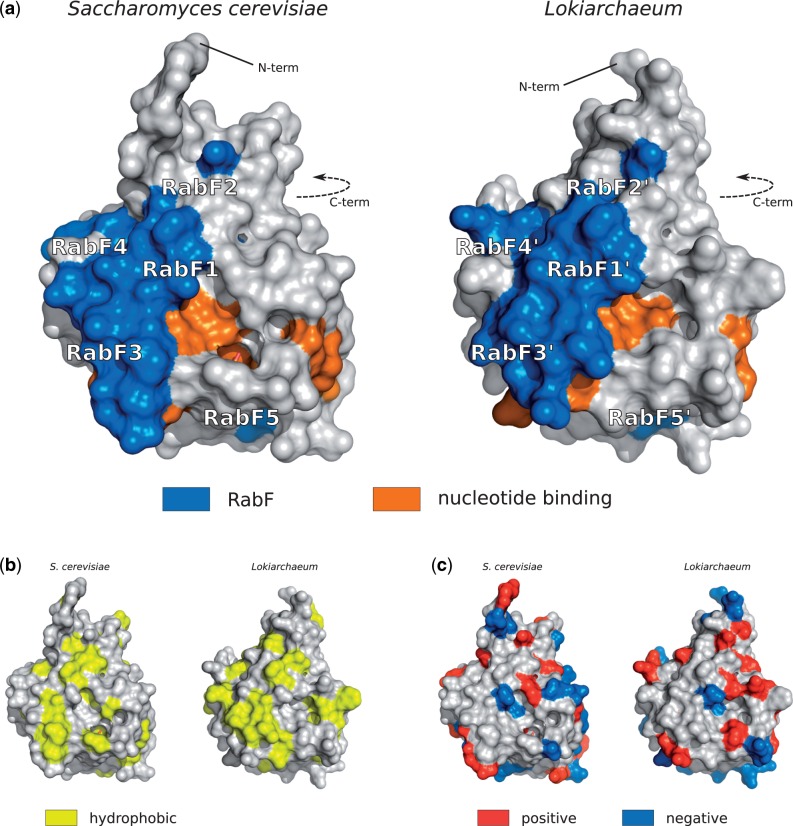
Structure comparison between yeast Ypt1 (left, PDB:1YZN) and a model of an archaeal Rab-like protein (right). (*a*) Location of RabF motifs and guanine nucleotide-binding motifs at the protein surface, (*b*) surface distribution of hydrophobic (Ala, Gly, Val, Ile, Leu, Phe, Met), and (*c*) charged residues (positively charged Arg, His, Lys and negatively Asp, Glu).

Given a high level of the primary sequence similarity between the archaeal Rab-like proteins and their eukaryotic counterparts, we modeled a putative 3D structure of a Rab-like GTPase and compared the location of Rab-specific features at the structural level. We chose a Lokiarchaeum sequence that contains all five RabF motifs (GenBank:KKK40223), as predicted by the Rabifier. To ensure a high quality of the model, we selected four templates from different Rab subfamilies that both have a high level of sequence identity to the archaeal homologue and a good crystallographic resolution of the 3D structure: Rab8 (*H. sapiens*, PDB:4LHW), Rab26 (*H. sapiens*, PDB:2G6B), Rab30 (*H. sapiens*, PDB:2EW1), and Ypt1 (*Saccharomyces* *cerevisiae*, PDB:1YZN). All template structures were in the active state, that is, bound to a GTP molecule. We used Modeller (Sali and Blundell 1993), a homology modeling platform to predict a putative structure of the archaeal protein (using all four templates simultaneously) and subsequently assessed its quality and stability. We obtained a similar structure using Phyre2 (Kelley et al. 2015), an automatic server for protein structure prediction and analysis (not shown). Figure 4 shows structures of both the model and the yeast template. Rab motifs are highlighted in blue (RabF motifs) and orange (guanine nucleotide-binding residues). Both structures are very similar (0.41 Å root-mean-square deviation of the C*α* atomic coordinates), motifs are localized at the same structural elements and similarly exposed to the environment. We also compared the location of hydrophobic (fig. 4b) and charged (fig. 4c) amino acids at the protein surface and observed a similar distribution of the residues in both structures.

We assessed the putative GTPase activity and the nucleotide-dependent conformational change of the archaeal Rab-like protein by analyzing its thermodynamic stability at both the GDP and GTP-bound state and predicting interactions between the protein and the phosphate groups of the nucleotide. In addition to the model of the GTP-bound state, we modeled the structure of the GDP-bound form, again using several templates belonging to different Rab subfamilies: Rab1 (*Cryptosporidium parvum*, PDB:2RHD), Rab2 (*H. sapiens*, PDB:2A5J), Rab8 (*H. sapiens*, PDB:4LHV), and Rab43 (*H. sapiens*, PDB:2HUP). The analysis of the structural predictions shows that the archaeal Rab-like protein is thermodynamically stable in both conformations (both predicted structures are shown in supplementary fig. S5, Supplementary Material online). The interaction between the phosphate groups and the protein is stabilized by several residues present in the protein active site. The presence of Gln68 and its relative position to the GTP molecule enables the interaction between a water molecule and the phosphate, necessary for the GTP hydrolysis (Dumas et al. 1999). The analysis of structural models of the archaeal Rab-like GTPase indicates that it can exist in two stable conformations and it is able to cycle between an “on” and “off” state like other small GTPases and, in particular, eukaryotic Rabs.

### A Rab Escort Protein/GDP Dissociation Inhibitor Ancestor in Archaea

**Fig. 5:**
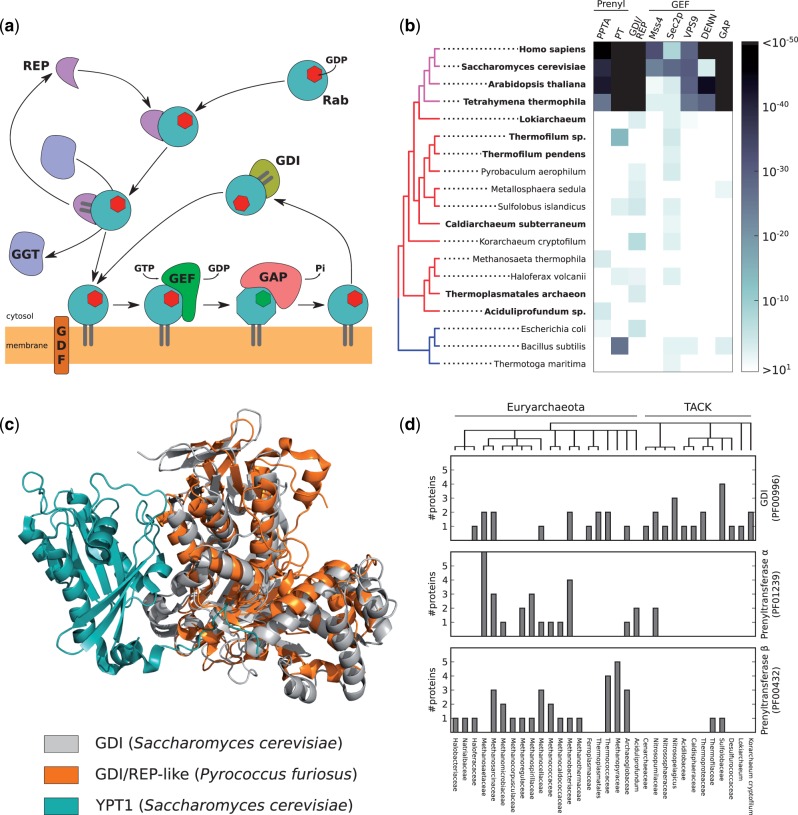
Identification of Rab regulatory proteins. (*a*) Schematic representation of the Rab activation pathway. (*b*) Homology detection by the similarity search of structural domains characteristic to the Rab regulatory proteins. Numbers represent e-values of the best scoring proteins in a species for each domain. Bold font indicates species predicted to contain Rab or Rab-like GTPases. GEF, guanine nucleotide exchange factor; GAP, GTPase-activating protein; GDI, GDP dissociation inhibitor; GGT, geranylgeranyl transferase; GDF, GDI displacement factor; REP, Rab escort protein; PPTA, Protein prenyltransferase alpha subunit; PT, Prenyltransferase. (*c*) Structural alignment of GDI/REP-like proteins from *Pyrococcus furiosus* and a GDI-YPT1 complex from *Saccharomyces cerevisiae*. (*d*) Total number of proteins containing the prenylation complex domains encoded in a genome. Each archaeal family is represented by a species with the biggest number of proteins containing selected domains. Tree topology is consistent with NCBI Taxonomy.

Our analysis so far suggests that Rab-like sequences predate Eukaryogenesis. Surprisingly, we found motifs in archaeal Rab-like sequences that are known to mediate interactions between eukaryotic Rabs and their regulators and effectors. Eukaryotic Rabs are prenylated on the C-terminus, a posttranslational modification catalyzed by the enzyme Rab geranylgeranyltransferase, which requires a chaperone termed REP (Rab Escort Protein) (Pereira-Leal et al. 2001; Leung et al. 2006); a paralogue of REP, termed GDI (GDP dissociation Inhibitor) recycles Rabs in and out of membranes (Wu et al. 1996; fig. 5a). Binding of Rabs to REP and GDI is mediated by residues in the RabF motifs (Rak et al. 2003, 2004; Goody et al. 2005). The same regions are involved in binding other general Rab regulators—Rab activity is regulated by guanine-nucleotide-exchange factors (GEF) that turn Rabs “on” by promoting the GDP to GTP exchange, and by GTPase-activating proteins (GAP) that increase GTP hydrolysis rate and turn Rabs off. Both sets of proteins interact with Rabs with residues included in the RabF motifs (those within the switch regions). The identification of RabF motifs in Archaea raises the hypothesis that such proteins and interactions could also predate Eukaryogenesis.

We used two approaches to test if homologues of these eukaryotic proteins can be detected in Archaea, indicating that some of the complex Rab regulatory cycles could predate Eukaryogenesis. First, we used sequences of several human regulators (GEFs, GAPs, FNT, PGGT1B, REP, RABGGT), performed BLAST (Altschul et al. 1990) similarity searches against archaeal genomes and found only hits with insignificant sequence similarity (not shown). As BLAST is known to lack sensitivity to detect remote homologies, we then used a more sensitive approach based on pHMM. We retrieved pHMMs (Pfam) of the domains that are found in Rab-binding proteins (Mss4, Sec2, VPS9, DENN, RabGAP-TBC, GDI/REP, prenyltransferase, PPTA), which we then used as queries for a similarity search using the HMMER package. In most cases, we found only scattered hits on the tree with marginal sequence similarity (fig. 5b), suggesting that either canonical Rab regulatory proteins are absent from Archaea or their sequences diverged from the eukaryotic counterparts beyond the detection level of standard automated methods. In one case, however, that of REP/GDI, even though the statistics of the hits were poor, we observed repeated positive hits, which we then investigated further.

We manually inspected putative GDI/REP domains in Archaea. The primary sequence of GDI and REP domain containing proteins is generally weakly conserved in Eukaryotes, both within each family and between GDI-REP paralogues (e.g., 30% human and fruit fly REP, 21% human GDI1 and REP1, local alignment identity). Hence, given the evolutionary distance between Eukaryotes and Archaea we expect that any putative archaeal homologs would be within the “twilight zone” of sequence similarity, which precludes any automatic sequence-based analysis. We used a fold recognition method (Jones 1999) with the best scoring (HMMER) archeal GDI/REP protein to detect candidate proteins with determined 3D structures. The best predictions belong to eukaryotic GDIs and archaeal proteins without experimentally determined function (top three hits correspond to proteins from *Bos taurus* PDB:1D5T, *Pyrococcus furiosus* PDB:3NRN, and *S.* *cerevisiae* PDB:2BCG). These structures are also very similar to FAD-containing monooxygenases and oxidases (Schalk et al. 1996), including archaeal geranylgeranyl reductases. While the sequence identity between putative archaeal GDI/REP and eukaryotic GDI is very low, at the structural level both domains (3NRN and 1UKV, a yeast GDI in complex with YPT1) are similar, including the Rab-binding platform (fig. 5c); our structural comparison revealed several residues that may form interactions with Rab switch regions (not shown). Our results strongly support the existence of a REP/GDI-like molecule in the TACK group, whose function implies an isoprenyl-binding ability.

We further used the same strategy to investigate whether the isoprenylation machinery, specifically the two subunits (*α* and *β*) of the eukaryotic isoprenyl transferases, is present in Archaea. Both approaches were inconclusive to determine the existence of the *α* subunit, as the tetratricopeptide repeat that characterizes this domain is widespread and functionally promiscuous, precluding any conclusion about function. However, we detected archaeal proteins whose predicted fold matches several isoprenoid metabolism enzymes including the geranylgeranyl transferase subunit *β*. We found multiple instances of genes containing these domains, observing some species where they co-occur (fig. 5d).

## Discussion

In this work, we investigated the hypothesis that the separation of the eukaryotic signature Rab sequences predates the emergence of Eukaryotes. This hypothesis follows from the recent discovery of a new archaeal group, the Lokiarchaeota, that was claimed to be a sister group of Eukaryotes. Our Rabifier pipeline identified 42 candidate Rab-like sequences that have multiple features related to eukaryotic Rabs, they exist in several Archaea of both the TACK group and Euryarchaeota but are particularly abundant in Lokiarchaeum. Although phylogenetic methods alone were insufficient to determine the position of Rab-like proteins within the RAS superfamily, our results indicate that these GTPases may be Rab precursors. Surprisingly, we also found evidence for a GDI/REP-like protein existing in Archaea, raising the possibility that this interaction predates Eukaryogenesis.

Small GTPases are well known to exist in prokaryotes, where they mediate diverse functions, for example, MglA regulates cell polarity and motility by accumulating at a cell pole in its active GTP-bound state (Zhang et al. 2010). The closest group to eukaryotic Rab/Rho/Ras/Ran are the Rup proteins (Ras superfamily GTPase of unknown function in prokaryotes; Wuichet and Søgaard-Andersen 2015). Phylogenetic analysis is not able to resolve the relationship between eukaryotic small GTPases and prokaryotic ones, so no claim can be made whether these sequences are Rup-like or a new independent branch (supplementary fig. S6, Supplementary Material online).

We concentrated on characterizing sequence and structural features that could shed light on the relationship between these sequences and eukaryotic Rabs. At the family level, they are more similar to the Rab family than to other eukaryotic small GTPases (Arf/Ras/Rho/Ran). We found extensive RabF motifs conservation, motifs that in Eukaryotes are diagnostic of this family, and that mediate important protein interactions characteristic of Rabs. On the structural models of archaeal Rab-like proteins, these motifs map to the same positions as their eukaryotic counterparts, suggesting that they could mediate similar interactions, which lends further support to their Rab-like classification. Our results thus point to Archaea having Rab-like sequences, which although not being full-fledged Rabs, as we will discuss below, are already differentiated intermediates to this small GTPase family.

The presence of Rab motifs that are known to mediate interactions with other Eukaryote-specific Rab regulators was puzzling and led us to test the hypothesis that one or more of these interactions could have predated eukaryogenesis. Using sensitive methods we found convincing REP/GDI-like proteins in multiple Archaea that are involved in the biosynthesis of membrane lipids (geranylgeranyl reductase, EC 1.3.1.101). An archaeal form of this enzyme had its crystal structure solved and aligns well with the crystal structure of GDI:Rab complex. It is thus very probable that the conservation of the RabF motifs in archaeal Rab-like sequences points to an established interaction with this enzyme. The functional meaning of this interaction is unclear, but the fact that this enzyme is involved in the synthesis of the isoprenoids that are used in the lipid modification of eukaryotic small GTPases is highly suggestive. Inspection of the structure of the archaeal enzyme suggests that although it has a binding pocket able to shield the lipid groups from the cytosol as REP and GDI do, it is in a different orientation, suggesting that it cannot chaperone lipid-modified eukaryotic Rabs that have longer C-termini than the archaeal Rab-like sequences.

In Eukaryotes REP/GDI are chaperones of the lipid-modified Rabs, that deliver them to the membranes, where REP is doing so in the context of the lipid modification reaction, as an accessory protein to the RabGGTase complex, and where GDI recycles Rabs in and out of membranes. The presence of a REP/GDI homologue in Archaea raises the hypothesis that membrane association of small GTPases via prenylation may have preceded the emergence of Eukaryotes. There is, at least, one report claiming isoprenylation of proteins in Archaea (Konrad and Eichler 2002). However, the absence of an extended C-terminal region beyond the GTPase globular domain together with the absence of the prenylateable C-terminal cysteine residues points against this. Furthermore, we found no evidence of a polybasic region that is known to mediate membrane association (Williams 2003), nor of any other membrane association signal. Our results thus suggest that these Rab-like sequences are unlikely to associate with membranes via lipidation. It is, however, interesting to note that archaeal homologues of both the alpha and beta subunits of eukaryotic prenyltransferases are common, although there is no evidence that they are able to form a heterodimer with the prenyltransferase activity. The beta subunit homologues are involved in the isoprenoid metabolism and their structure is predicted to be similar to eukaryotic prenyltransferases, which further supports the notion that some components of the prenylation complex are present in Archaea.

Small GTPases are molecular switches that can cycle between two membrane-associated states, as well as cycle in an out of membrane. Our results suggest that these Archaea represent a snapshot of the evolution of this circuit, that resolves part of the evolutionary path into membrane-associated protein trafficking regulators. The Rab protein family is already individualized, even though we lack any known internal membranes in the TACK Archaea. These proteins are apparently active GTPases able to cycle between two structural states, but it is unclear if they do it in the cytosol or if an “in” and “out” of membrane switch was already established. In this scenario, an interaction with the protein that will become the chaperone that catalyses this second part of the Rab cycle is already established, but in the absence of lipid modification. It is plausible that localization to membranes may exist via protein-protein interactions. Finally, the building blocks for a protein prenylation machinery are also found in multiple Archaea, suggesting that even the emergence of this component of the Rab cycle may also predate eukaryogenesis.

Our conclusions are possible because we were able to go beyond phylogenetic methods, which are clearly insufficiently sensitive to resolve events at this order of temporal divergence, using instead our motif/domain-based tool to identify Rabs, the Rabifier. It is important now to look into other small GTPase families, as our preliminary data suggest that other members of the Ras/Rho/Ran/Rab clade may have already been individualized in Archaea. It is also important to investigate whether the interaction we predict here between Rab-like and REP/GDI-like sequences does in fact exist, and what is the subcellular localization of these small GTPases. Lokiarchaeota, are unlikely target organisms for these experiments, as they exist in a difficult to reach environment. However, organisms that are routinely cultured in the laboratory have these sequences (see fig. 5), which makes these experiments tractable. Furthermore, we found that other environmental (marine) samples (Kawai et al. 2014) also possess Lokiarchaeota-like small GTPases and specifically abundant Rab-like sequences (117 proteins in the analyzed sample), which makes the possibility of isolation and culture of these organisms more plausible. Our study gives further support to the notion that Eukarya emerged from within Archaea, and may be construed to support the notion that it was from within organisms close to the recently identified Lokiarchaeum. We are convinced that in the near future we will be able to resolve the origin of the in-out of membrane cycle of small GTPases, and their association with specific eukaryotic processes. It is possible that this cycle emerged in Archaea, even before the specific system they regulate in Eukaryotes has emerged, and that have later been co-opted.

## Materials and Methods

### Sequences

All complete archaeal proteomes (231) were downloaded from the UniProt database (The UniProt Consortium 2015), all Lokiarchaeum proteins (5,384) were downloaded from GenBank (Benson et al. 2014). The complete list of species is shown in the supplementary table S1, Supplementary Material online. Eukaryotic and bacterial genomes were downloaded from Ensembl (Cunningham et al. 2015).

### Protein Sequence Alignments

Multiple sequence alignments were built with MAFFT 7.221 (Katoh and Standley 2013) using a high accuracy mode (–maxiterate 1,000 –localpair). TrimAl v1.2 (Capella-Gutiérrez et al. 2009) was used to remove gap-rich regions from alignments. Pairwise sequence alignments were constructed with water (the Smith–Waterman local alignment algorithm) and needle (the Needleman–Wunsch global alignment algorithm) from the EMBOSS package (Rice et al. 2000). Jalview 2.8.2 (Waterhouse et al. 2009) was used for alignment visualization.

### Phylogeny Reconstruction

Phylogeny reconstruction using the Bayesian inference was conducted with MrBayes 3.2.5 (Ronquist et al. 2012) using the mixed amino acid model with gamma-distributed rate variation across sites. Two parallel runs with four chains each (Metropolis coupling) were run until the topologies converged (standard deviation of split frequencies is below 0.05), first 25% generations were discarded as the burn-in. RAxML 8.1.22 (Stamatakis 2014) was used for tree reconstruction using the maximum likelihood method, a discrete approximation to the gamma distribution with four categories was used to model across-site rate heterogeneity, the best-fitting substitution model (LG, Le and Gascuel 2008) was selected using ProtTest 3.4 (Darriba et al. 2011). ETE2 (Huerta-Cepas et al. 2010) and Dendroscope3 (Huson and Scornavacca 2012) were used for tree visualization.

### Sequence Analysis

pHMMs of protein families were build from sequence alignments using hmmbuild from the HMMER 3.1b2 software package (http://hmmer.org, last accessed April 6, 2016), plurality-rule consensus sequences were generated with hmmemit. Sequence logos were generated with WebLogo 3.4 (Crooks et al. 2004) from multiple sequence alignments.

Amino acid variation was calculated for each position in an alignment of paralogous proteins as the entropy of that position, $H(X)=-\sum_{i=1}^{n}p(x_{i})\log_{2}p(x_{i})$, where $p(x_{i})$ is the fraction of the residue *x_i_* at the *X* column in the alignment.

### Protein Structure Prediction

MODELLER v9.15 (Sali and Blundell 1993), a program which implements a homology-based method for structure modeling, was used to predict protein structures given templates with known structure that share a high level of sequence identity to the modeled protein. Model quality and stability were evaluated with the DOPE potential (Shen and Sali 2006), ProSA (Sippl 1993; Wiederstein and Sippl 2007), and Verify3D (Lüthy et al. 1992). PyMOL (The PyMOL Molecular Graphics System, Version 1.7.4 Schrödinger, LLC.) was used for structure visualization.

## Supplementary Material

Supplementary figures S1–S6 and tables S1–S3 are available at *Molecular Biology and Evolution* online (http://www.mbe.oxfordjournals.org/).

Supplementary Data
