# Supplementary material for: Are There Rab GTPases in Archaea?
Source: Mol Biol Evol. 2016 Mar 31;33(7):1833–42. doi: 10.1093/molbev/msw061 (PMC4915359; doi:10.1093/molbev/msw061)
Supplement: Supplementary Data [file supp_33_7_1833__index.html]

Are There Rab GTPases in Archaea? — Are There Rab GTPases in Archaea? — Supplementary Data 

# Are There Rab GTPases in Archaea?

## Supplementary Data

files

- Supplementary Data - pdf file
