# Supplementary material for: Are There Rab GTPases in Archaea?
Source: Mol Biol Evol. 2016 Mar 31;33(7):1833–42. doi: 10.1093/molbev/msw061 (PMC4915359; doi:10.1093/molbev/msw061)
Supplement: Supplementary Data [file supp_msw061_SupplementaryMaterial.pdf]

Supplementary material

Table S1. List of archaeal genomes

Taxon ID	Name
2162	Methanobacterium formicicum
2209	Methanosarcina mazei
2287	Sulfolobus solfataricus
28892	Methanofollis liminatans DSM 4140
43687	Metallosphaera sedula
64091	Halobacterium sp. NRC-1
69014	Thermococcus kodakarensis KOD1
70601	Pyrococcus horikoshii OT3
79929	Methanothermobacter marburgensis str. Marburg
113653	Geoglobus ahangari
163003	Thermococcus cleftensis
178306	Pyrobaculum aerophilum str. IM2
186497	Pyrococcus furiosus DSM 3638
187420	Methanothermobacter thermautotrophicus str. Delta H
188937	Methanosarcina acetivorans C2A
190192	Methanopyrus kandleri AV19
192952	Methanosarcina mazei Go1
195522	Thermococcus nautili
213585	Methanosarcina mazei S-6
224325	Archaeoglobus fulgidus DSM 4304
224719	Methanobrevibacter sp. AbM4
228908	Nanoarchaeum equitans Kin4-M
243232	Methanocaldococcus jannaschii DSM 2661
246969	Thermococcus sp. AM4
259564	Methanococcoides burtonii DSM 6242
261388	Ferroplasma sp. Type II
261391	Thermoplasmatales archaeon Gpl
263820	Picrophilus torridus DSM 9790
267377	Methanococcus maripaludis S2
268739	Natronomonas moolapensis 8.8.11
269797	Methanosarcina barkeri str. Fusaro
272557	Aeropyrum pernix K1
272569	Haloarcula marismortui ATCC 43049
272844	Pyrococcus abyssi GE5
273057	Sulfolobus solfataricus P2
273063	Sulfolobus tokodaii str. 7
273075	Thermoplasma acidophilum DSM 1728
273116	Thermoplasma volcanium GSS1
304371	Methanocella paludicola SANAE
309800	Haloferax volcanii DS2
311458	Candidatus Caldiarchaeum subterraneum
323259	Methanospirillum hungatei JF-1
330779	Sulfolobus acidocaldarius DSM 639
333146	Ferroplasma acidarmanus fer1
339860	Methanosphaera stadtmanae DSM 3091
340102	Pyrobaculum arsenaticum DSM 13514
342949	Pyrococcus sp. NA2
348780	Natronomonas pharaonis DSM 2160
349307	Methanosaeta thermophila PT
351160	Methanocella arvoryzae MRE50
362976	Haloquadratum walsbyi DSM 16790
368407	Methanoculleus marisnigri JR1
368408	Thermofilum pendens Hrk 5
374847	Candidatus Korarchaeum cryptofilum OPF8
384616	Pyrobaculum islandicum DSM 4184
387631	Archaeoglobus sulfaticallidus PM70-1
391623	Thermococcus barophilus MP
397948	Caldivirga maquilingensis IC-167
399549	Metallosphaera sedula DSM 5348
399550	Staphylothermus marinus F1
402880	Methanococcus maripaludis C5
406327	Methanococcus vannielii SB
406552	Natrinema sp. J7-2
410358	Methanocorpusculum labreanum Z
410359	Pyrobaculum calidifontis JCM 11548
414004	Cenarchaeum symbiosum A
415426	Hyperthermus butylicus DSM 5456
416348	Halorubrum lacusprofundi ATCC 49239
419665	Methanococcus aeolicus Nankai-3
419942	Sulfolobus islandicus Y.N.15.51
420247	Methanobrevibacter smithii ATCC 35061
425944	Sulfolobus islandicus L.D.8.5
426118	Sulfolobus islandicus M.16.4
426368	Methanococcus maripaludis C7
427317	Sulfolobus islandicus M.14.25
427318	Sulfolobus islandicus M.16.27
429572	Sulfolobus islandicus L.S.2.15
436308	Nitrosopumilus maritimus SCM1
439386	Sulfolobus islandicus Y.G.57.14
439481	Aciduliprofundum boonei T469
444157	Pyrobaculum neutrophilum V24Sta
444158	Methanococcus maripaludis C6
453591	Ignicoccus hospitalis KIN4/I
456320	Methanococcus voltae A3
456442	Methanoregula boonei 6A8
469382	Halogeometricum borinquense DSM 11551
478009	Halobacterium salinarum R1
485914	Halomicrobium mukohataei DSM 12286
490899	Desulfurococcus kamchatkensis 1221n
519442	Halorhabdus utahensis DSM 12940

521011	Methanosphaerula palustris E1-9c
523841	Haloferax mediterranei ATCC 33500
523846	Methanothermus fervidus DSM 2088
523849	Thermococcus litoralis DSM 5473
523850	Thermococcus onnurineus NA1
529709	Pyrococcus yayanosii CH1
543526	Haloterrigena turkmenica DSM 5511
547558	Methanohalophilus mahii DSM 5219
547559	Natrialba magadii ATCC 43099
555311	Sulfolobus solfataricus 98/2
565033	Geoglobus acetivorans
572478	Vulcanisaeta distributa DSM 14429
572546	Archaeoglobus profundus DSM 5631
573063	Methanocaldococcus infernus ME
573064	Methanocaldococcus fervens AG86
579137	Methanocaldococcus vulcanius M7
582419	Thermococcus paralvinellae
583356	Ignisphaera aggregans DSM 17230
589924	Ferroglobus placidus DSM 10642
591019	Staphylothermus hellenicus DSM 12710
593117	Thermococcus gammatolerans EJ3
593750	Methanoregula formicica SMSP
604354	Thermococcus sibiricus MM 739
633148	Thermosphaera aggregans DSM 11486
634497	Haloarcula hispanica ATCC 33960
634498	Methanobrevibacter ruminantium M1
644281	Methanocaldococcus sp. FS406-22
644295	Methanohalobium evestigatum Z-7303
647113	Methanothermococcus okinawensis IH1
666510	Acidilobus saccharovorans 345-15
667135	Thermoplasmatales archaeon A-plasma
667137	Thermoplasmatales archaeon E-plasma
667138	Thermoplasmatales archaeon I-plasma
673860	Aciduliprofundum sp. MAR08-339
679901	Methanosalsum zhilinae DSM 4017
679926	Methanolacinia petrolearia DSM 11571
693661	Archaeoglobus veneficus SNP6
694429	Pyrolobus fumarii 1A
694430	Natronococcus occultus SP4
698757	Pyrobaculum oguniense TE7
751944	Halobacterium sp. DL1
756883	halophilic archaeon DL31
765177	Desulfurococcus mucosus DSM 2162
768065	Haloquadratum walsbyi C23
768672	Desulfurococcus fermentans DSM 16532
768679	Thermoproteus tenax Kra 1
795797	Halalkalicoccus jeotgali B3
797210	Halopiger xanaduensis SH-6
797299	Halostagnicola larsenii XH-48
797302	Halovivax ruber XH-70
797303	Natrinema pellirubrum DSM 15624
797304	Natronobacterium gregoryi SP2
866790	Methanothermobacter sp. CaT2
867904	Methanomethylovorans hollandica DSM 15978
868131	Methanobacterium paludis
877455	Methanobacterium lacus
880724	Methanotorris igneus Kol 5
886738	Candidatus Nitrosoarchaeum limnia SFB1
926571	Nitrososphaera viennensis EN76
930943	Sulfolobus islandicus HVE10/4
930945	Sulfolobus islandicus REY15A
933801	Acidianus hospitalis W1
937775	Methanoplanus limicola DSM 2279
985053	Vulcanisaeta moutnovskia 768-28
990316	Methanosaeta concilii GP6
999630	Thermoproteus uzoniensis 768-20
1006006	Metallosphaera cuprina Ar-4
1028566	Sulfolobus acidocaldarius N8
1028567	Sulfolobus acidocaldarius Ron12/I
1033806	Halorhabdus tiamatea SARL4B
1041930	Methanocella conradii HZ254
1042877	Thermococcus sp. 4557
1053692	Methanococcus maripaludis X1
1054217	Thermoplasmatales archaeon BRNA1
1056495	Caldisphaera lagunensis DSM 15908
1094980	Methanolobus psychrophilus R15
1104324	Pyrobaculum sp. 1860
1110509	Methanosaeta harundinacea 6Ac
1163730	Fervidicoccus fontis Kam940
1183377	Pyrococcus sp. ST04
1184251	Thermogladius cellulolyticus 1633
1185654	Pyrococcus furiosus COM1
1198449	Aeropyrum camini SY1 = JCM 12091
1201294	Methanoculleus bourgensis MS2
1229908	Candidatus Nitrosopumilus koreensis AR1
1229909	Candidatus Nitrosopumilus sp. AR2
1236689	Candidatus Methanomethylophilus alvus Mx1201
1236903	Methanosarcina mazei Tuc01
1237085	Candidatus Nitrososphaera gargensis Ga9.2
1241935	Sulfolobus islandicus LAL14/1
1262903	Methanoculleus sp. CAG:1088
1263088	Methanobrevibacter smithii CAG:186
1295009	Candidatus Methanomassiliicoccus intestinalis Issoire-Mx1
1301915	Methanocaldococcus sp. JH146
1333523	Salinarchaeum sp. Harcht-Bsk1
1343739	Palaeococcus pacificus DY20341
1344584	Archaeoglobus fulgidus DSM 8774
1365176	Thermofilum sp. 1910b
1379702	Methanobacterium sp. MB1

1410573	uncultured Acidilobus sp. MG
1410574	uncultured Acidilobus sp. CIS
1410575	uncultured Acidilobus sp. OSP8
1410576	uncultured Acidilobus sp. JCHS
1410606	Candidatus Nitrosopelagicus brevis
1417673	Haloarcula hispanica N601
1434099	Methanosarcina sp. Kolksee
1434100	Methanosarcina sp. MTP4
1434102	Methanosarcina sp. WH1
1434103	Methanosarcina sp. WWM596
1434104	Methanococcoides methylutens MM1
1434106	Methanosarcina barkeri 227
1434107	Methanosarcina barkeri 3
1434108	Methanosarcina barkeri MS
1434109	Methanosarcina barkeri str. Wiesmoor
1434110	Methanosarcina horonobensis HB-1 = JCM 15518
1434111	Methanosarcina lacustris Z-7289
1434113	Methanosarcina mazei C16
1434114	Methanosarcina mazei LYC
1434115	Methanosarcina mazei SarPi
1434117	Methanosarcina mazei WWM610
1434118	Methanosarcina siciliae C2J
1434119	Methanosarcina siciliae HI350
1434120	Methanosarcina siciliae T4/M
1434123	Methanosarcina vacuolata Z-761
1459636	Candidatus Nitrososphaera evergladensis SR1
1483596	Methanosarcina sp. 2.H.T.1A.15
1483597	Methanosarcina sp. 2.H.T.1A.3
1483598	Methanosarcina sp. 2.H.T.1A.8
1483599	Methanosarcina sp. 2.H.T.1A.6
1483600	Methanosarcina sp. 2.H.A.1B.4
1483601	Methanosarcina sp. 1.H.A.2.2
1483602	Methanosarcina sp. 1.H.T.1A.1
1505907	Thermococcus eurythermalis
1538547	archaeon Loki
1577791	Candidatus Methanoplasma termitum
1579370	archaeon GW2011_AR10
1579373	archaeon GW2011_AR15
1579378	archaeon GW2011_AR20
1580092	Candidatus Nitrosopumilus sp. NF5
1582439	Candidatus Nitrosopumilus sp. D3C
1592728	Haloarcula sp. CBA1115
1609232	Thermoproteus sp. AZ2

Table S2. Family consensus sequence identity (lower triangle, gray background) and similarity (upper triangle) calculated using the Smith-Waterman local alignment algorithm.

	Rab	Rab-like	Ran	Rho	Ras	Arf
Rab		0.78	0.57	0.58	0.62	0.48
Rab-like	0.60		0.59	0.56	0.67	0.56
Ran	0.34	0.34		0.46	0.54	0.42
Rho	0.39	0.38	0.28		0.54	0.43
Ras	0.41	0.42	0.31	0.37		0.46
Arf	0.29	0.33	0.29	0.23	0.29	

Table S3. Family consensus sequence identity (lower triangle, gray background) and similarity (upper triangle) calculated using the Needleman-Wunsch global alignment algorithm.

	Rab	Rab-like	Ran	Rho	Ras	Arf
Rab		0.71	0.53	0.56	0.60	0.41
Rab-like	0.55		0.47	0.52	0.57	0.51
Ran	0.32	0.26		0.41	0.47	0.30
Rho	0.37	0.35	0.25		0.51	0.38
Ras	0.40	0.36	0.27	0.35		0.39
Arf	0.25	0.30	0.20	0.20	0.24	

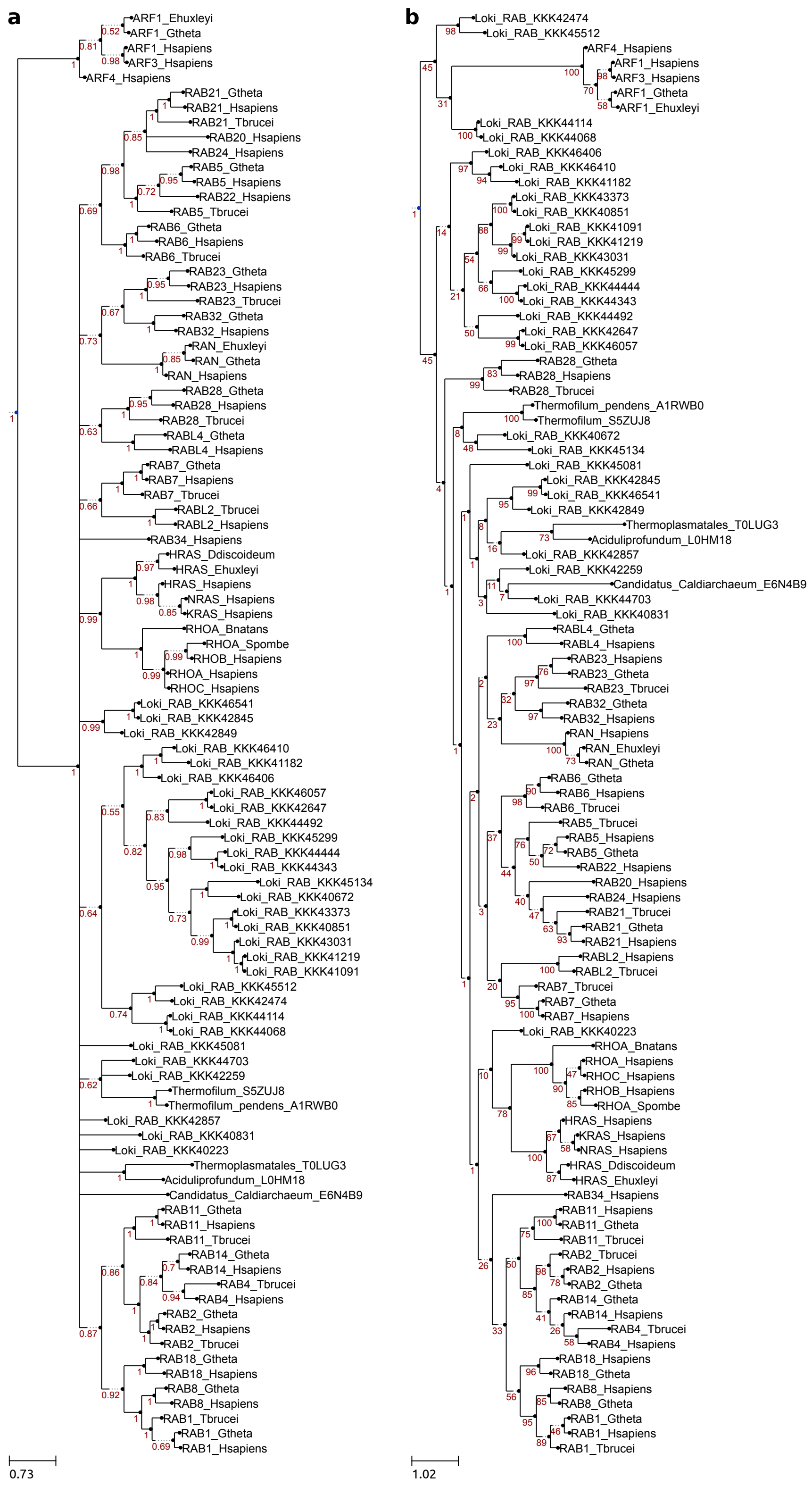

Fig. S1. (a) Bayesian phylogeny of Eukaryotic and Archaeal small GTPases with MrBayes (mixed model, across-site rate heterogeneity, 5000000 generations). (b) Maximum likelihood phylogeny of Eukaryotic and Archeal small GTPases estimated using RAxML with GAMMALG model, branch support was estimated with 1000 rapid bootstraps. Branch lengths are proportional to the expected number of substitutions per site, as indicated by the scale bar.

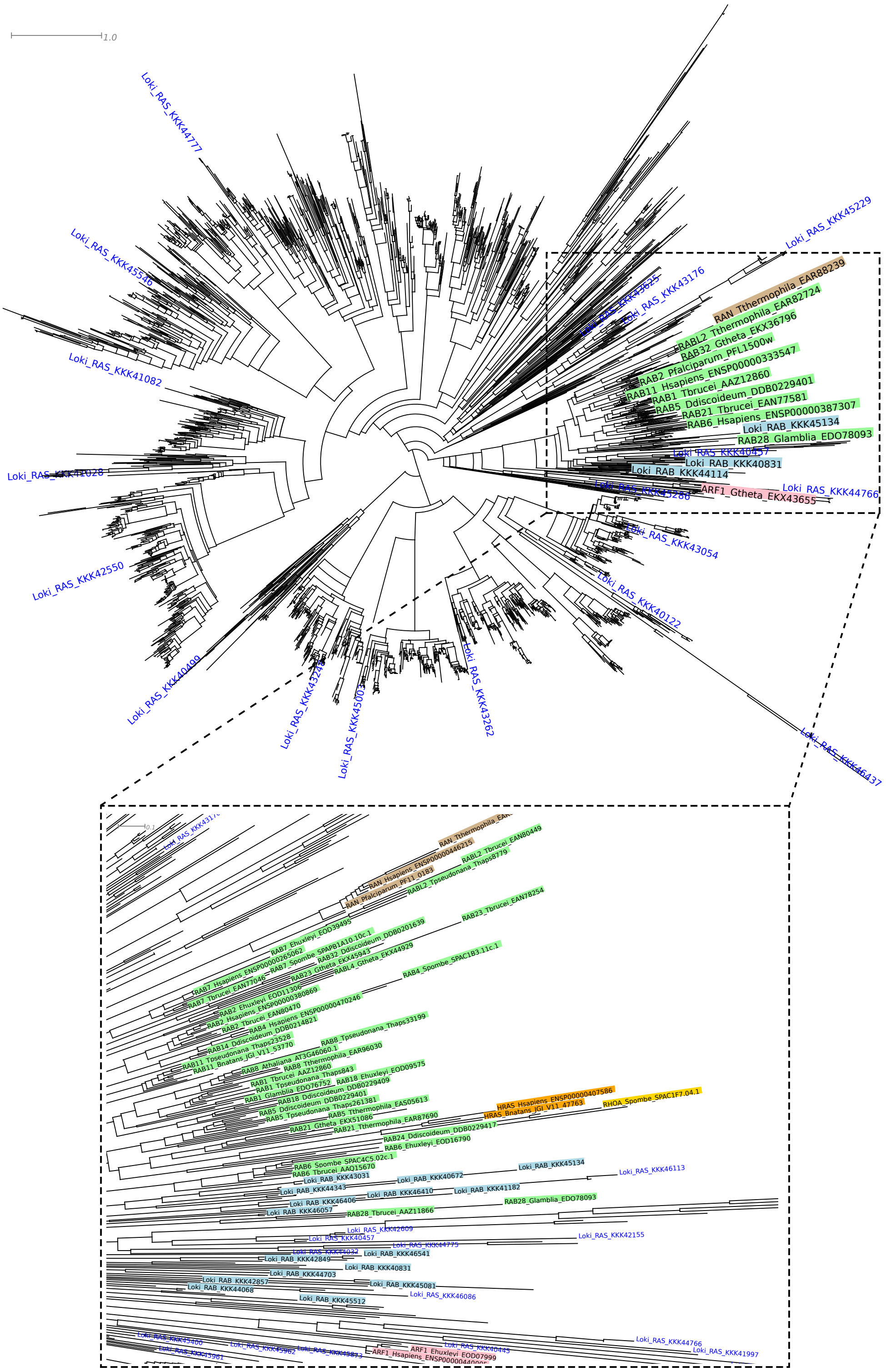

Fig. S2. Maximum likelihood phylogeny of all archeal small GTPases and representatives from major eukaryotic RAS families. RAxML with GAMMALG+F model was used to estimate the maximally supported tree. Proteins: 120 eukaryotic Rab, 23 other eukaryotic RAS, 35 putative Archeal Rabs, 2315 other small GTPases from Archaea.

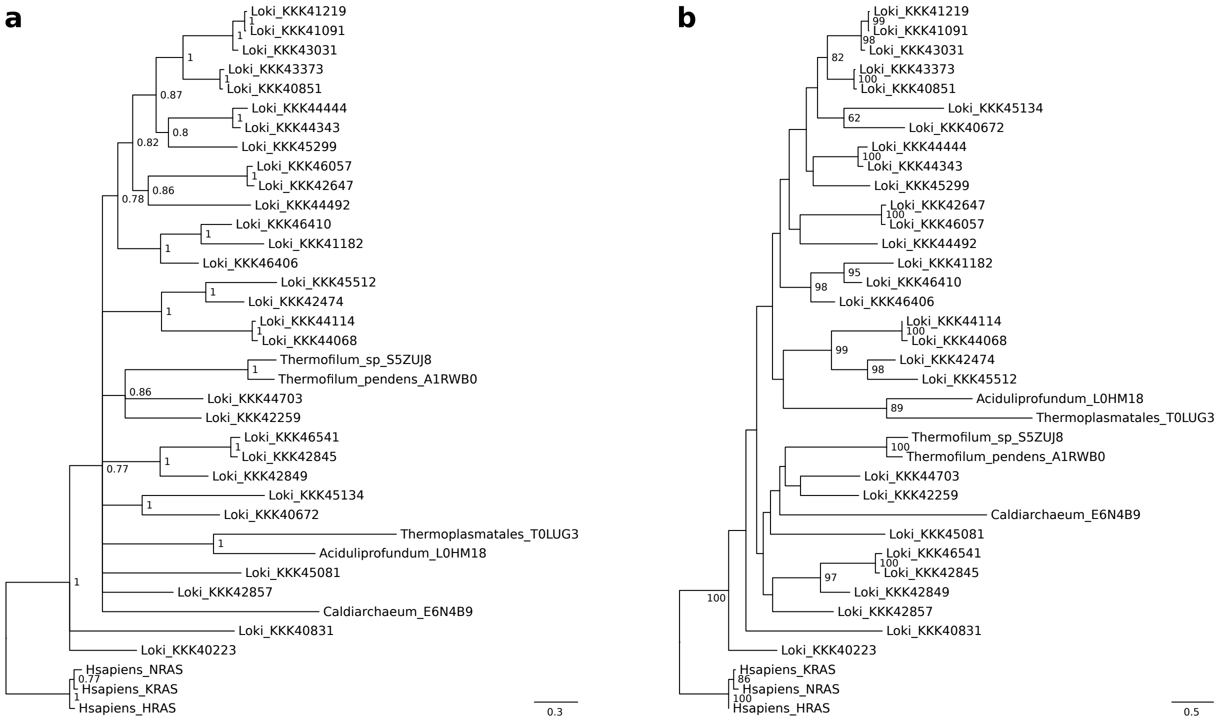

Fig. S3. Phylogenetic tree constructed using the Bayesian (a) and maximum likelihood (b) inference of archaeal Rab-like proteins, members of the human Ras family were used as an outgroup. Branch support is given with the Bayesian posterior probability (a) and bootstrap value (b). Branch lengths are proportional to the expected number of substitutions per site, as indicated by the scale bar.

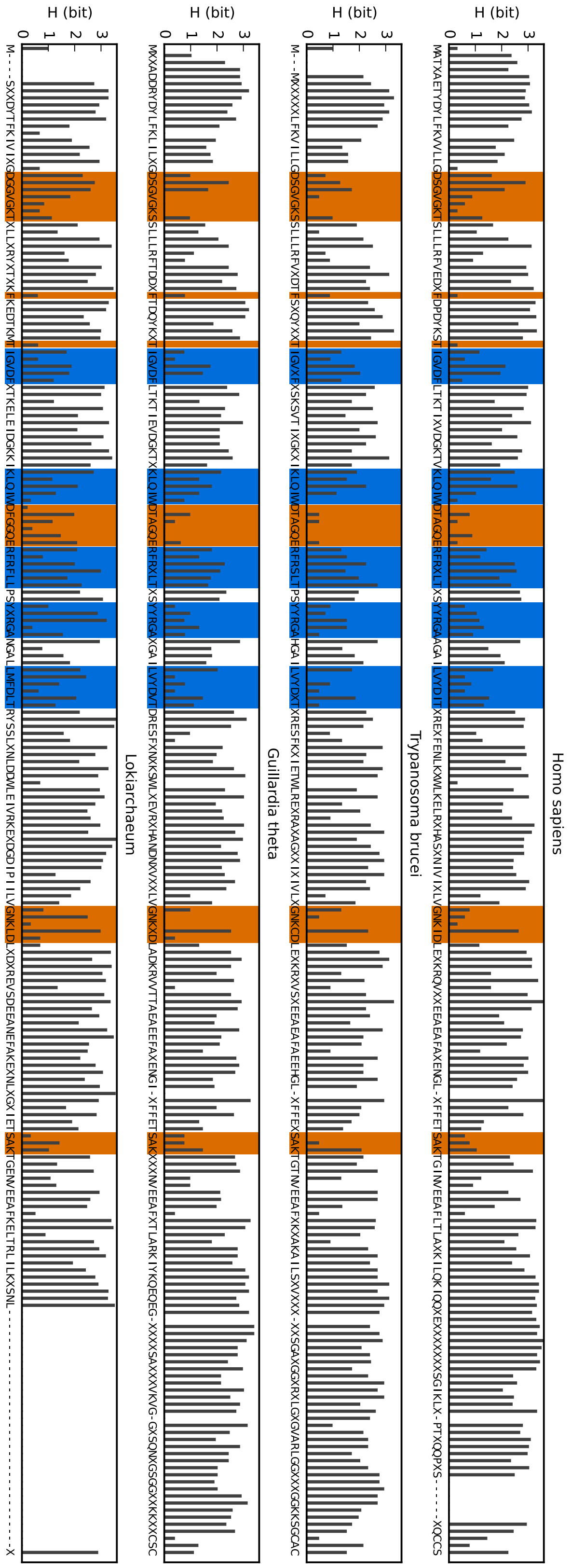

Fig. S4. Sequence variation (H, entropy) across Rab paralogues in four species. Sequence were aligned for each species, amino acid variation was estimated for each column in the alignment and consensus sequence was calculated (X denotes positions where the frequency of the most common amino acid is lower than 0.2). Plot shows the alignment of consensus sequences and sequence variation at each site.

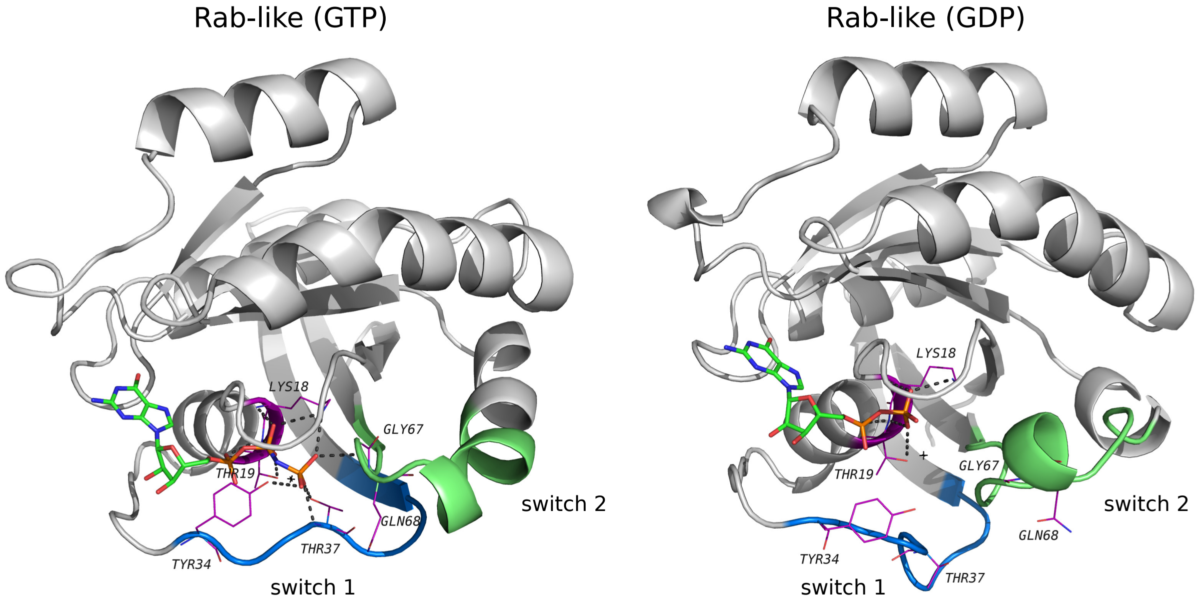

Fig. S5. Archaeal Rab-like protein in the GTP and GDP-bound form.

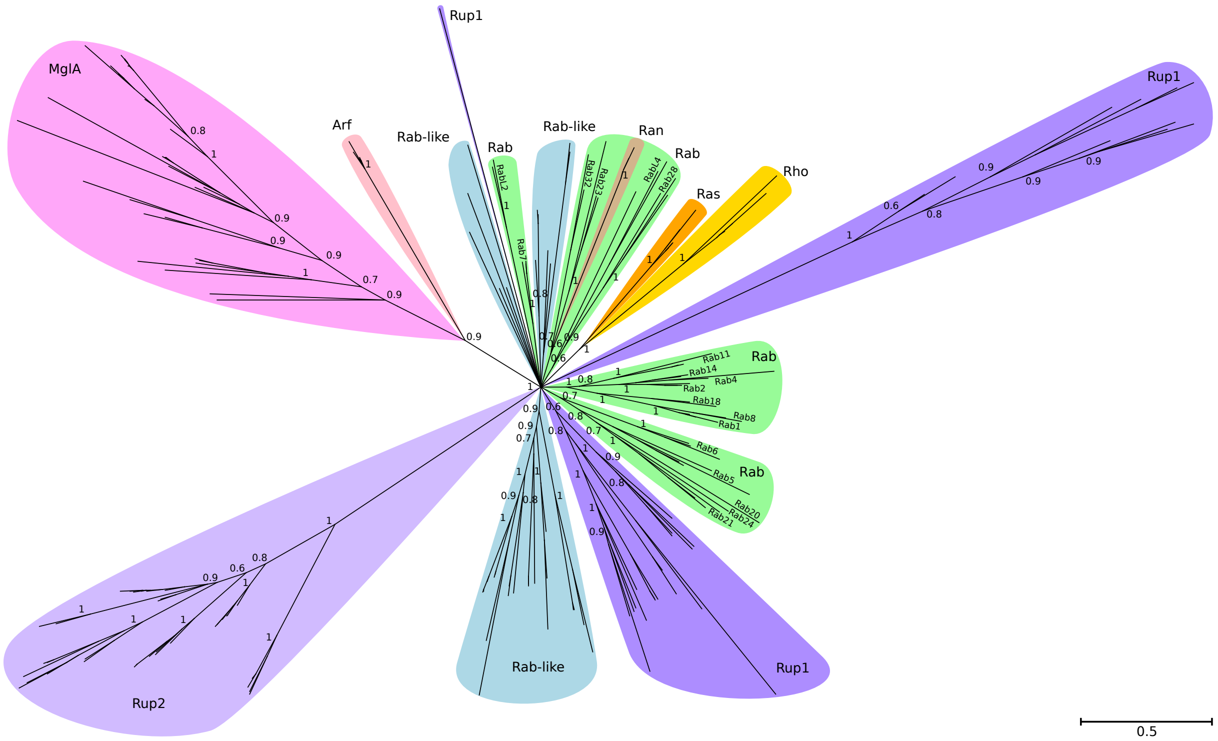

Fig. S6. Bayesian phylogeny of several small GTPase families from Archaea and Eukaryotes. Branch lengths are proportional to the expected number of substitutions per site, as indicated by the scale bar.
